# Retraction Note: Maslinic acid induces anticancer effects in human neuroblastoma cells mediated via apoptosis induction and caspase activation, inhibition of cell migration and invasion and targeting MAPK/ERK signaling pathway

**DOI:** 10.1186/s13568-026-02107-4

**Published:** 2026-08-25

**Authors:** Yusheng Liu, Hongting Lu, Qian Dong, Xiwei Hao, Lingyan Qiao

**Affiliations:** 1 Department of Pediatric Surgery, The Affiliated Hospital of Qingdao University, Shibei District, No. 6, Tongfu Road, Qingdao, 266000 Shandong China; 2 Department of Pediatric Endocrinology and Genetic Metabolic Diseases, Qingdao Women and Children’s Hospital, Qingdao, 266000 Shandong China

**Retraction Note to: AMB Expr (2020) 10:104** 10.1186/s13568-020-01035-1

The Editor-in-Chief has retracted this article. Concerns were raised regarding a number of figures, specifically:

- Figure 3: sections of the 0 µM and 10 µM panels appear to be similar, after a rotation and change in scale;
- Figure 4: sections of all panels appear to be similar to Figure 2 of [1] and Figure 2A of [2], after rotations and changes in scale;
- Figure 5: the 10 µM, 40 µM, and 80 µM panels appear to be similar to Figure 4 of [3] and Figure 5 of [4], after changes in scale;
- Figure 8: the 0 µM, 40 µM, and 80 µM panels appear to be similar to Figure 9 of [5], Figure 9 of [6], and Figure 4A of [7], after changes in scale. Sections of the 0 µM and 10 µM panels appear to be similar, after a change in scale;
- Figure 9: the 40 µM panel appears to be similar to a panel of Figure 4B of [8], after rotation and a change in scale. Sections of the 0 µM and 10 µM panels appear to be similar.

The authors provided some source data on request from the Publisher, but this was insufficient to address the concerns raised. The Editor-in-Chief therefore no longer has confidence in the reliability of the data reported in the article.

The authors have not responded to any correspondence from the Publisher regarding this retraction.

